# Novel Technologies for the detection of Fusarium head blight disease and airborne inoculum

**DOI:** 10.1007/s40858-017-0138-4

**Published:** 2017-02-14

**Authors:** Jonathan S. West, Gail G. M. Canning, Sarah A. Perryman, Kevin King

**Affiliations:** Rothamsted Research, Harpenden, Hertfordshire AL5 2JQ UK

**Keywords:** Disease risk, Spore-trap, qPCR, LAMP, Optical sensing

## Abstract

Many pathogens are dispersed by airborne spores, which can vary in space and time. We can use air sampling integrated with suitable diagnostic methods to give a rapid warning of inoculum presence to improve the timing of control options, such as fungicides. Air sampling can also be used to monitor changes in genetic traits of pathogen populations such as the race structure or frequency of fungicide resistance. Although some image-analysis methods are possible to identify spores, in many cases, species-specific identification can only be achieved by DNA-based methods such as qPCR and LAMP and in some cases by antibody-based methods (lateral flow devices) and biomarker-based methods (‘electronic noses’ and electro-chemical biosensors). Many of these methods also offer the prospect of rapid on-site detection to direct disease control decisions. Thresholds of spore concentrations that correspond to a disease risk depend on the sampler (spore-trap) location (whether just above the crop canopy, on a UAV or drone, or on a tall building) and also need to be considered with weather-based infection models. Where disease control by spore detection is not possible, some diseases can be detected at early stages using optical sensing methods, especially chlorophyll fluorescence. In the case of *Fusarium* infections on wheat, it is possible to map locations of severe infections, using optical sensing methods, to segregate harvesting of severely affected areas of fields to avoid toxins entering the food chain. This is most useful where variable crop growth or microclimates within fields generate spatially variable infection, i.e. parts of fields that develop disease, while other areas have escaped infection and do not develop any disease.

## Introduction

In many parts of the world, head blight (ear blight or scab) in small grain cereals is caused by a complex of species in the genus *Fusarium*, *Giberella* and *Microdochium*. It is important to know which head blight pathogens are attacking crops as there may be important differences in host resistance, toxin production or sensitivity to fungicides that will affect control decisions or end-use of the affected crop. Often, the species present in a region and their relative abundance is affected by the local climate, types of crops grown and other agricultural practices, which affect inoculum production and infection success (Del Ponte et al. 2009; O’Donnell et al. 2004; Qiu and Shi 2014). Climate change is also likely to exacerbate the disease by increasing inoculum production and dispersal and also by reducing host resistance (Vaughan et al. 2016). Species of current concern in north-western Europe are deoxynivalenol (DON) toxin producers, *F. culmorum* and *F. graminearum sensu stricto*, against which no wheat variety is fully resistant. Wheat grains are only susceptible to infection for a relatively short period during flowering up to the early dough stage (Del Ponte et al. 2007) and infection is encouraged by warm springs and rainfall around anthesis (West et al. 2012). In addition, increased maize cultivation and a trend towards non-inversion or minimal-tillage has increased inoculum availability because the crop residues persist for a long time and produce large amounts of inoculum (Blandino et al. 2010). The timing of airborne inoculum risk and presence of suitable infection conditions can be predicted using within-season weather-based disease forecasting models (De Wolf et al. 2003; Hooker et al. 2002; Moschini et al. 2001). Ideal conditions for infection are 25°C and 100% RH for 24 hr post (Abramson et al. 1987; Parry et al. 1995). However, variation in the response of the pathogen to weather events and differences between microclimates in the crop canopy and soil surface can cause uncertainty in predictions of inoculum availability. For air-dispersed pathogens like *F. graminearum*, it is possible to use air sampling to produce a direct warning of inoculum presence, which reduces uncertainty of predictions. A range of air sampling methods are reviewed below with various pathogens used as examples. In addition, optical sensing, including remote sensing from satellites, aircraft and drones (UAVs) or ground-based vehicles, is another useful method for detection and mapping of disease symptoms. In the case of head blight, it is not possible to control the disease once symptoms have appeared but symptom detection can help with later decisions on the harvesting and end-use of the grain and is also of use for phenotyping of host material in disease resistance breeding trials. Various methods are reviewed below.

## Air sampling for enhanced disease control

Air sampling has been used to study the epidemiology of crop diseases in order to design a control strategy – how to alter time of sowing or harvest to escape disease (Davidson et al. 2013), when precisely to apply fungicides or other crop protection agents (Brachaczek et al. 2016; Cao et al. 2016; Carisse et al. 2009; Thiessen et al. 2016; West et al. 2002), and whether it is possible to separate susceptible crops from inoculum sources (Maldonado-Ramirez et al. 2005; Marcroft et al. 2003). Spore sampling is useful to monitor changes in pathogen populations for both fundamental research and applied purposes such as providing a direct forecast of imminent disease risk by detecting the inoculum before infection events start.

**Fig. 1:**
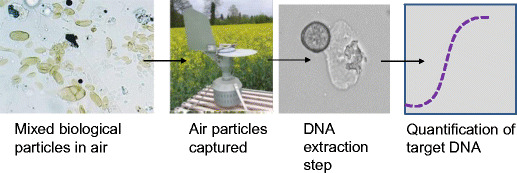
Stages in the detection of a plant pathogen from airborne samples

Air particulate samples are typically a deposit of dust, spores, pollen, plant fragments and other microscopic material, either captured by impaction onto a sticky surface, or collected by devices that produce a rapid change of direction of airflow, which causes particles to be deposited onto a surface or into a tube (West and Kimber 2015). Until the early 2000s, samples were routinely identified by microscopy. This lengthy and skilled process is often only possible for identification of relatively large and visually characteristic spores because spores of many species look very similar or even identical and cannot be identified to the species level with high certainty. As a result, a range of diagnostic methods have been applied to air-particulate samples. Many recent advances in spore samplers have been designed to improve the ease of sampling and to enhance the downstream application of diagnostic methods to the sample. In the past 15 years or so, most spore samples have been processed not for microscopy but have DNA extracted from the mixture of particles and then tested by PCR or qPCR to detect a specific target organism e.g. (Carisse et al. 2009; Gent et al. 2009; Kunjeti et al. 2016); (Fig. 1).

For lab-based studies, qPCR is often preferred as this method produces accurate quantification allowing the amount of pathogen DNA detected to be translated into a number of spores present in the air sample and therefore the concentration of spores per cubic metre of air sampled. DNA-based diagnostics can be designed in some cases to detect specific genotypes of a species such as a race that can infect a certain crop variety (Kaczmarek et al. 2014) or a mutant that is resistant to a class of fungicide. Already DNA of the air spora (i.e. spores and other biological particles in the air) has been sequenced but the process is currently relatively expensive, although costs are reducing (personal communication, Mogens Nicolaisen, December 2016, re: article submitted).

Other isothermal DNA-based diagnostics such as LAMP and TwistDX are now being used as they may be more rapid than PCR, use less-expensive equipment or be less prone to inhibition by chemicals present naturally at times in some of the components of the air spora (Hansen et al. 2016; Thiessen et al. 2016). These isothermal DNA amplification methods offer the prospect of samples to be analysed using relatively simple equipment and even in the field. It is possible to use these methods not only for pathogen detection but even quantification of specific pathogen DNA onsite in a matter of minutes. Sophisticated immunological diagnostics and biosensors can also perform this role relatively cheaply and rapidly (Wakeham et al. 2016).

The optimal deployment of air samplers varies according to how widespread or common the pathogen is, the volume of air sampled by the device used and the importance or value of the crop (West and Kimber 2015). Inoculum-based forecasts are best suited to sporadic but damaging diseases of crops and particularly those that infect at early or later crop growth stages (such as Fusarium) when farmers do not routinely spray. In particular, diseases that cause yield-loss in high-value crops such as vegetables or fruits, are also likely to benefit from inoculum-based disease forecasting. It is also not cost-effective to sample for exotic or unusual pathogens that only cause epidemics very rarely (Madden et al. 2007).

Thresholds of spore concentrations to trigger disease control operations are difficult to define and are different for each pathogen. Spore concentrations in air decline with distance from the source and height above the ground. If the exact location of the source is not known, we cannot infer how much dilution has occurred ahead of the sample being taken, i.e. a relatively high concentration of spores in the sample could be caused by a small source of spores very close to the sampler, or by a very large distant source. Some smoothing or buffering against the effects of proximity of the sampler to the source can be achieved by mounting air samplers on the roof of a tall building or on a drone or UAV. The decrease in signal strength due to dilution can be a problem for roof-top sampling but it can also be counter-acted by the air being better mixed, representing the air spora released from a wider range of microclimates present in the environment upwind of the sampling position. Therefore, a single air sampler located at rooftop height can be used to infer the presence of common plant pathogen airborne inoculum over a regional scale. However, for many sporadic pathogens a denser network of samplers is needed to provide the full picture of inoculum distribution. Further research is needed to understand issues of the spatial variability in spore concentrations and how that relates to subsequent disease risk. The prospect of automated samplers that are networked to send near real-time data could then provide a new aspect of precision agriculture – knowing exactly where and when to apply crop protection agents.

## Optical sensing for disease management

Diseases can cause a range of different responses in plants from changes in leaf colour, shape or size to disturbances to the plant’s photosynthesis, transpiration, canopy morphology and canopy density (West et al. 2003). This alters the thermal and optical properties of the plant sufficiently for remote sensing to be used to quantify and even map symptoms. With the case of Fusarium head blight and wheat blast, it should be possible to map affected locations of fields to allow segregation of more severely affected parts of fields or entire fields from areas that are relatively unaffected. This can occur due to slight differences in crop growth stage in different parts of fields or nearby entire fields, causing some of the crop to escape disease, while other parts can be severely affected.

**Fig. 2:**
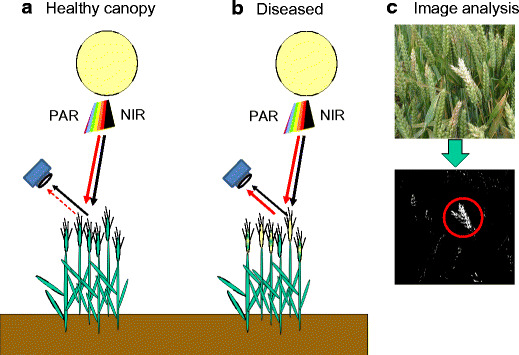
Illustration of a camera recording light reflected from a healthy or disease-affected canopy. The quality of light from the healthy canopy has a low ratio of photosynthetically active radiation (PAR) to Near InfraRed (NIR), while this ratio is increased in the diseased canopy. Indices such as normal differential vegetation index (NDVI) can be used to improve classification of affected crops. A traditional camera needs to take duplicate images with different filters in front of the lens to allow this processing for classification of disease but modern hyperspectral or multispectral digital cameras can filter light reaching individual pixels of the image sensor surface, allowing images in different wavebands to be taken simultaneously, which can be processed using a computer. Such cameras can be smaller than a tennis ball enabling them to fit onto a drone (UAV). Alternatively, Fig. 2c shows processing of an image of Fusarium affected wheat from a standard digital camera into a simple black and white image, from which clusters of white pixels over a chosen threshold size can indicate an infected location

Generally, differences in the appearance of healthy and diseased plants can be detected simply by spectroscopy, which measures light quality in the ‘field of view’ of a sensor, by sensing the intensity of wavebands either over the entire spectrum or in just selected specific wavebands. Alternatively, differences in reflectance caused by diseases can be distinguished more powerfully using an imaging system to produce a focussed image that can be analysed pixel by pixel for differences in intensity at specific wavebands (Fig. 2). Different broad wavebands that can be useful for discriminating plant health are summarised in Table 1 (Carter 1993; Moshou et al. 2011; Rouse et al. 1973; West et al. 2003).

**Table 1:** Comparison of thermal and optical features indicative of healthy and diseased crops

Waveband	Diagnostic ability
Visible (400–700 nm)	Increased reflectance in diseased canopies, especially in the chlorophyll absorption bands due to loss of chlorophyll and presence of surface spores or mycelium
Near Infra-Red (NIR 700-1200nm)	The red edge (rapid transition from low reflectance to high reflectance) is shifted from 730 nm in healthy canopies to shorter wavelengths (e.g. 670 nm) in diseased canopies. Reflectance is also reduced in diseased canopies due to senescence and defoliation.
Short-wave Infra-Red (SWIR 1200–2400 nm)	Relatively small effects occur due to water content
Thermal infrared band (TIR ≈ (8000–14000 nm)	Leaf temperature is increased by reduced transpiration rate, caused by root diseases and wilts (xylem infection) and some foliar diseases (that cause closure of stomata at early stages). Water-soaked leaf lesions (caused by cell lysis) can appear cooler at the start of the day or warmer at the end of the day as the rest of the leaf changes temperature more quickly according to ambient conditions.

To develop a method to measure crop disease, it is important to validate the method on a wide range of symptom severities and under a diverse variety of conditions such as leaf wetness, types of cloud cover and solar angle or ambient light quality. For both spectroscopy and imaging, practical systems can measure just a few discriminatory wavebands, identified by prior research for a particular disease or type of symptom. These can be processed to reduce data storage, e.g. by dividing the intensity of light at one wavelength with that at another wavelength to make an index. A common index is NDVI or Normalised Differential Vegetation Index, which is an indicator of how much green leaf area is present. NDVI is calculated from the intensity of light measured in just two wavebands, near-infrared (NIR; waveband 780–890 nm) and red (R; waveband 650–680 nm) using the equation: (NIR—R) / (NIR + R) (Table 2).

**Table 2:** Common Vegetation indices used for plant disease detection

Index	Name	Formula based on reflectance of given bands or wavelengths (nm)	Reference
DVI	difference vegetation index	NIR - R	Tucker and Red (1979)
RVI	ratio vegetation index	NIR / R	Jordan (1969)
NDVI	normalized differential vegetation index	(NIR-R) / (NIR + R)	Rouse et al. (1974)
GNDVI	Green normalized difference vegetation index	index (NIR - G) / (NIR + G)	Gitelson et al. (1996)
NBNDVI	Narrow-band normalized difference vegetation index	(850–680) / (850 + 680)	Thenkabail et al. (2000)
NRI	Nitrogen reflectance index	(570—670) / (570 + 670)	Filella et al. (1995)
TVI	Triangular vegetation index	0.5[120(750–550) - 200(670–550)]	Broge and Leblanc (2001)
P/PRI	Photochemical/Physiological Reflectance Index	(531–570) / (531 + 570)	Gamon et al. (1992)
TCARI	The transformed chlorophyll absorption and reflectance index	3[(700–670) - 0.2(700–550)(700 / 670)]	Haboudane et al. (2004)
MCARI	Modified chlorophyll absorption ratio index	[(701–671)- 0.2(701–549)] / (701/671)	Daughtry et al. (2000)
RVSI	Red-Edge Vegetation Stress Index	[(712+752)/2] - 732	Merton and Huntington (1999)
PSRI	Plant Senescence Reflectance Index	(680–500)/750	Merzlyak et al. (1999)
ARI	Anthocyanin Reflectance Index	(550)-1 - (700)-1	Gitelson et al. (2001)
λ_red_	Red edge position	Wavelength at red edge	Gong et al. (2002)
dr_red_	Red edge slope	Maximum value of 1st derivative with in red edge	Gong et al. (2002)
Σdr_680–760 nm_	The area of the red edge peak	The area under the derivative curve in the region of red edge	Gong et al. (2002)

R = Reflectance of red band within wavelengths 650 to 680 nm

NIR = Reflectance of near-infrared band within wavelengths 780 to 890 nm

G = Reflectance of green band within wavelengths 560 to 600 nm.

Using an index avoids measuring every wavelength of the spectrum, which would create a massive dataset. Many other indices can be used - common ones are listed in Table 2, adapted from Cao et al. (Cao et al. 2015).

Spectroscopy and imaging methods can each be used to map diseases and other stresses in fields. Platforms for sensors can include tractors, UAVs, aircraft or satellites. More proximal platforms such as tractors can produce image pixel sizes <1 mm^2^, while satellite images have pixel sizes of 1–400 m^2^. Satellite images are therefore best for scouting problems over large areas and usually are not able to identify the cause of the problem. Proximal sensors (e.g. tractor-mounted) are better than remote sensors to differentiate disease symptoms from symptoms of nutrient-stress (West et al. 2003). Artificial supplementary light can improve data collection, particularly if powerful visible or UV light is applied to induce fluorescence of cell walls and chlorophyll, which often can indicate altered photochemical efficiency of a plant before disease symptoms become visible (Cecchi et al. 1994; Scholes 1992; Wright et al. 1995). Fluorescence imaging typically uses cameras fitted with bandwidth filters or multispectral cameras (which have filters over individual pixels of the image sensor surface) to allow fluorescence in different wavebands to be used for diagnosis.

The cost of thermal imaging has been reducing considerably with tiny cameras now available cheaply that can be used with a Raspberry Pi computer. Since changes in the rate of transpiration (caused by early senescence) or disruption of root and xylem function, influence the temperature of leaves and ears in TIR wavebands (8000–14000 nm), it is possible to detect and map fusarium infection using thermal imaging (Oerke and Steiner 2010). In the case of fusarium head blight and wheat blast, this type of detection is only possible when it is too late for disease control options to be applied and is unlikely to allow discrimination of wheat blast and fusarium head blight without complicated pattern recognition methods being used. However, detection of symptoms can allow a more targeted sampling regime to assess which pathogen is present. In addition, where toxigenic *Fusarium* species are the main pathogen, optical sensing could allow segregation of harvesting severely-affected areas of fields from less-affected areas. It is also possible to use optical sensing to separate healthy and infected harvested grain (Barbedo et al. 2015).

## Conclusions

A new type of precision disease management is emerging based on using air sampling with DNA-based diagnostic methods. This can be automated and integrated with wireless communications along with weather data to drive infection models, which together can provide a warning of imminent disease risk. This could be applied to improve control of Fusarium in crops by allowing decisions to be made in time for crop protection products to be effective or to reduce costs of unnecessary applications. Additionally, positions of fields affected by fusarium head blight could also be mapped using reflectance or thermal imaging to allow segregation of harvested grain. Fluorescence can be useful to detect infection at early stages and appears to be a promising method for many diseases. This approach appears not to be appropriate for fusarium control in production field, however, as current fungicides are not effective once infection has become established. Despite this, optical sensing methods can be useful to quantify host-resistance phenotype in pre-breeding and commercial breeding trials. Improved crop protection is increasingly important, particularly for intensive production systems and helps to reduce the environmental impact per unit of produce harvested.
